# The NETest liquid biopsy is diagnostic for gastric neuroendocrine tumors: observations on the blood-based identification of microscopic and macroscopic residual diseaseOK

**DOI:** 10.1186/s12876-020-01348-2

**Published:** 2020-07-23

**Authors:** A. Malczewska, A. Procner, A. Walter, K. Kusnierz, W. Zajecki, H. Aslanian, B. Kos-Kudla

**Affiliations:** 1 Department of Endocrinology and Neuroendocrine Tumors, Medical University of Silesia, ul. Ceglana 35, 40-514 Katowice, Poland; 2 Department of Gastrointestinal Surgery, Medical University of Silesia, ul. Medykow 14, 40-752 Katowice, Poland; 3 Department of Pathology, Medical University of Silesia, ul. 3 Maja 13-15, 41-800 Zabrze, Poland; 4 Department of Digestive Diseases, Center for Advanced Endoscopy, Yale University School of Medicine, 310 Cedar Street, New Haven, CT 06510 USA

**Keywords:** Biomarker, ECL cell, Gastrin, Gastric, Gastroscopy, Neuroendocrine tumor, NET, NETest, Liquid biopsy, Proliferome

## Abstract

**Background:**

NETest, a novel multi-gene liquid biopsy has utility in neuroendocrine tumor (NET) diagnosis and identification of residual disease. We independently assessed utility of the NETest to diagnose gastric neuroendocrine neoplasms (GNENs) and identify micro- and macroscopic residual disease.

**Methods:**

Cohorts comprised histologically confirmed GNENs at biopsy, *n* = 46; GNETs Type 1: 42 (32 NET G1, 10 NET G2), a GNET Type 3: 1 well-differentiated NET G3, neuroendocrine carcinomas (NECs) (*n* = 3), and controls (*n* = 63). Disease status at sampling was assessed by gastroscopy, histology (resection margin [R] positivity of polypectomy or biopsy), EUS, CT or MRI, and/or ^68^Ga-DOTA-TATE PET/CT. Groups included image- (gastroscopy, EUS, and anatomical and/or functional imaging) positive or image negative disease. NETest assay by PCR (spotted plates, normal cut-off: 20). Data: mean ± SD.

**Results:**

*Disease extent*: Image-negative (*n* = 30) (21 R0, 9 R1); Image-positive, *n* = 16.

*Diagnosis:* NETest was increased in GNETs (23 ± 11) vs. controls (7 ± 4, *p* < 0.0001). In histology-positive, the NETest accuracy was 100% (25/25).

*Microscopic disease:* In image-negative but R1, NETest was elevated in 100% (9/9; 28 ± 9). Levels were elevated vs. controls (7 ± 4, *p* < 0.0001), or R0 (16 ± 11, *p* = 0.02). Eight of 21 R0, exhibited positive NETest.

*Macroscopic disease:* Gastric lesions were multiple: 38%, single: 62%, submucosal: 13%, or ulcerated: 13%. Lesions size was ≤5 mm (50%), > 5–9.9 mm (17%), 10–19.9 mm (17%), ≥20 mm (17%) [≥10 mm: 34%). The NETest accuracy was 100% (16/16). Levels (28 ± 7) were higher than controls (7 ± 4, *p* < 0.0001) or R0 (16 ± 11, *p* = 0.002) but not to R1 (28 ± 9, *p* = 0.5).

**Conclusions:**

NETest is diagnostic for gastric NETs. Elevated levels identify both microscopic and macroscopic residual disease. In histology/image-negative disease, elevated NETest may reflect early evidence of increased neuroendocrine gene expression of hypergastrinemia-induced neoplastic transformation of enterochromaffin-like (ECL) cells to tumor status. A sensitive liquid biopsy has utility in the management and surveillance of gastric NET disease.

## Background

Gastric neuroendocrine neoplasms (GNENs) comprise a heterogeneous group of neuroendocrine neoplasia deriving from gastric neuroendocrine cells. Their increasing incidence most likely represents the widespread use of endoscopy [1]. The majority (80%) of GNENs are enterochromaffin-like (ECL) cell derived, and are mainly localized to the gastric fundus and body [2]. The tumors have been classified into three subtypes based upon their distinct etiopathogenesis, gastrin-dependency, and pathobiological characteristics [1, 3, 4].

The most common, Type 1, usually occurs in older women (50–70 years) [5], in a setting of chronic atrophic gastritis type A (CAG-A) and hypergastrinemia (70–80%) [6]. This group generally follows an indolent, benign and relatively asymptomatic course. The overall metastatic rate is low, and has been correlated to lesion size, with 10 mm set as a cut-off, or deep muscularis propria invasion [1, 4, 7]. However, despite the overall low risk of metastasis, surveillance programs are mandated [1, 8]. This reflects the risk of lesion progression or recurrence and the potential to develop gastric adenocarcinoma [3, 4, 8]. In order to monitor GNETs, repeated endoscopy and biopsy are required. This is invasive and costly and reflects the fact that there is no accurate biomarker to monitor these tumors. An unmet need is therefore the identification of a blood biomarker that correlates with disease aggressiveness or progress and can be used for GNEN diagnosis and surveillance. Current biomarkers such as gastrin and chromogranin A (CgA) are largely ineffective [6]. For example, gastrin has low utility as a biomarker since secretion is elevated in low acid conditions like CAG, during persistent *Helicobacter pylori* infection [9], or through use of proton pump inhibitors (PPIs) for e.g., gastroesophageal reflux disease (GERD) [6]. High gastrin levels drive fundic ECL proliferation and concomitantly increase CgA levels, rendering the interpretation of elevated values of each as difficult [10].

Similarly, monitoring using conventional imaging (CT/MRI) and ^68^Ga-SSA PET/CT are also of very limited utility in the localized, small polypoid, Type 1 GNETs and is associated with exposure to radiation. Consequently endoscopy (gastroscopy with biopsy) has become the default option for diagnostic workup and long-term monitoring of GNETs [10]. This, however, represents a substantial financial burden for the healthcare system and exposes patients to uncomfortable invasive techniques over a repeated time period, with a consequent decrease in compliance. Overall, given the low malignancy risk for NET recurrence, the cost/benefit ratio of this strategy requires careful reconsideration. Endoscopy is critical to identify the risk for adenocarcinoma but molecular genomic stratification can provide adjunctive information to identify low and high risk groups.

The recent development of a multianalyte molecular signature (NETest) for neuroendocrine tumors raises the possibility of reconsideration of the “endoscopy for life” strategy. The gene signature was derived from an enteropancreatic cohort, and captures biological information pertinent to the diagnosis and management of both pancreatic and small bowel neuroendocrine disease [11–17]. It recently has demonstrated utility for lung NET diagnosis and monitoring [18–20].

Although GEP-NENs represent a heterogeneous group of neoplasms, they each derive from neuroendocrine cells which share a common genotype and are dispersed throughout the digestive system [21, 22]. GNENs are a component of GEP-NENs [2] and previous reports have suggested that the NETest signature may be valid for GNETs. Positive scores have been noted in the 10 patients that were included in other cohorts (typically 1 or 2 patients per study) [11, 12, 15, 17, 23, 24]. Based on these observations, we considered that the NETest signature would be effective in the identification of GNENs.

The current study was designed therefore to independently assess the accuracy of the NETest in GNENs firstly as a diagnostic and secondly as an indicator of residual disease. NETest results in GNENs were compared to controls and were correlated with disease extent as documented by imaging modalities (gastroscopy, EUS and anatomical and/or functional imaging) at the time of blood draw.

## Methods

### Strategy

**Fig. 1:**
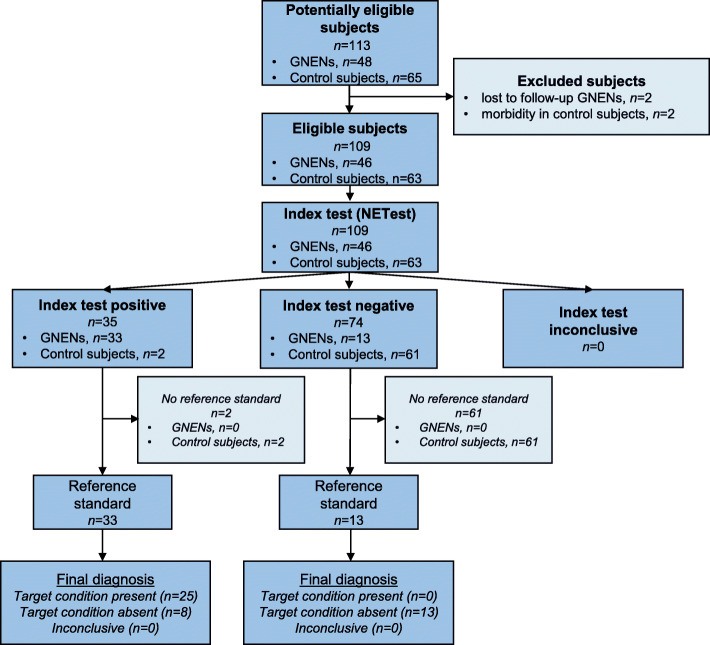
Standards for Reporting of Diagnostic Accuracy (STARD) flow chart. Index test is represented by the NETest assay undertaken in blood. Reference tests were the standard imaging and histological examinations in the follow-up of GNENs described in more detail in paragraphs: Disease evaluation by imaging and Histological evaluation

We examined circulating NETest levels from GNENs (*n* = 46) and compared these to controls (*n* = 63) using the STARD approach [25] (Fig. 1). The diagnostic accuracy and metrics (AUROC, sensitivity, specificity) for the NETest were calculated. Cohorts: histologically confirmed GNENs, *n* = 46; GNETs Type 1: 42 (32 NET G1, 10 NET G2), a GNET Type 3: 1 well-differentiated NET G3, poorly differentiated neuroendocrine carcinomas (NECs) (*n* = 3), and controls (*n* = 63). Disease status at sampling was assessed by gastroscopy and histology (resection margin [R] positivity of polypectomy or biopsy), EUS, CT or MRI, and/or ^68^Ga-DOTA-TATE PET/CT. Subjects were divided into groups: image modality (endoscopy/radiology)-positive (IPD) or image modality negative (IND) disease. The clinical data were collected retrospectively.

### Cohorts

The study was approved by the Institutional Ethics Committee of the Medical University of Silesia. Informed written consent was obtained from all study subjects. Blood samples were collected over a 15-month period between February 2017 and May 2018. Control subjects (*n* = 63) included healthy family members of hospital personnel, and non-affected family members of the patients attending endocrinology department. Control subjects were enrolled if there was no known malignancy at the time of blood draw and they identified themselves as asymptomatic and in good health. All NENs were histologically confirmed, with no other synchronous malignancy at blood draw.

Patient cohorts (GNENs, *n* = 46) are included in Table 1.

**Table 1:** Clinical characteristics of the study cohort

Variable	Category	GNET Type 1	GNEN G3	Controls
*Number*	No.	42	4	63
*Gender*	Males	10	3	20
	Females	32	1	43
*Age*	Mean (range)	55 (28–87)	55 (40–84)	44 (23–78)
*Age at initial diagnosis*	Mean (range)	53 (27–81)	51 (38–83)	N/A
*Follow-up since initial diagnosis (years)*	Mean (range)	2.6 (0.12–14.7)	3.8 (0.5–10.5)	N/A
*Grade*	Grade 1	32	–	N/A
	Grade 2	10	–	
	Grade 3 NET	–	1	
	NEC	–	3	
*Ki-67 (%)*	Mean (range)	2.5 (1–17)	51 (30–75)	N/A
*Active Chronic Atrophic Gastritis*	% (N)	81% (34)	N/A	N/A
*Gastrin Levels [N ≤ 115 pg/ml]*	% Elevated	75%^a^	25%^b^	N/A
	Mean FC (range)	4.2 (0.12–13.3)	0.57 (0.1–1.04)	
	Mean Value (range)	483 (18–1525)	66 (12–120)	
*Intestinal Metaplasia*	No.	31	0	N/A
*Proton pump inhibitor treatment*	% On treatment	7%	25%	
*Chromogranin A [N < 100μg/l]*	% Elevated	44%	25%	N/A
	Mean (range)	142 (20–700)	203 (20–700)	
*Disease extent by imaging and histology*	Image-positive & R1	15	1	N/A
	Image-negative & R0	18	3	
	Image-negative & R1	9	–	
*Current treatment*	Type	No (Surveillance)	No (Surveillance)	N/A
*Previous treatments*	None	0	1	N/A
	Polypectomy	24	0	
	Partial gastrectomy	7	1	
	Total gastrectomy	3	2	

N: within normal range; No.: number of cases; N/A: Not applicable; FC: Fold change; ^a^ 2 subjects on PPI; ^b^ hypergastrinemia on PPI; R – resection/polypectomy margin; Chromogranin A assay: ELISA (Tecan Sunrise, Austria); Gastrin Levels [N ≤ 115 pg/ml]

GNETs Type 1: 42 (32 NET G1, 10 NET G2), all sporadic and non-functioning. The mean follow-up time from initial diagnosis was 2.6 years (0.1–14.7). At the time of blood draw, active CAG was confirmed in 34 (81%). Gastrin levels were elevated in 75% (two subjects on PPI). Prior to referral to our center, seven individuals underwent partial gastrectomy for GNETs (4 NET G1, 3 NET G2), and three total gastrectomy (all NET G2, Ki-67 ≤ 10%). The partial gastrectomy subjects comprised 4 NETs G1 with Ki-67 of 1–2%, which ranged in size 0.7–4 cm, and for the NETs G2, tumor size ranged 8–11 mm and Ki-67’s ranged 3–10%. The partial gastrectomies were undertaken between 2006 and 2017 due to large polyps invading beyond muscularis propria, locoregional involvement, endoscopic submucosal dissection (ESD) complicated with bleeding, previous history of multiple instances of disease recurrence, and ulcerated submucosal lesions. The three total gastrectomy patients underwent the procedures for multiple, large - up to 6 cm - recurring polyps throughout the stomach not adequately controlled by either somatostatin analogues or previous polypectomy, Ki-67 between 3 and 10%, and evidence of invasive disease (infiltration of muscularis propria, and in two lymph node involvement was identified).

GNET Type 3 (*n* = 1): 1 well-differentiated NET G3; an in situ 3-cm gastric polyp was present. CAG was not identified. Gastrin levels were “mildly” elevated (120 pg/ml [ULN 115]) while on PPI.

GNECs (*n* = 3): All three GNECs were image modality-negative; 2 underwent total gastrectomy, and 1 partial gastrectomy. The mean follow-up since surgery was 4.8 years (1.7–10.5). CAG was not identified in these subjects. Gastrin levels were within normal range.

### Disease evaluation by imaging (endoscopy and anatomical and/or functional modalities)

Imaging modalities included endoscopy (gastroscopy), EUS, anatomical and/or functional. Image-positive was defined as lesion detection by any of these methods. Image-negative reflected the no detection by any of the above.

GNETs Type 1 (*n* = 42): Subjects were evaluated by upper endoscopy (*n* = 38), EUS (*n* = 15), CT (*n* = 24), MRI (*n* = 5), ^68^Ga-DOTA-TATE PET/CT (*n* = 26), or ^18^F-FDG PET/CT (*n* = 1).

GNET Type 3 (*n* = 1): The subject was evaluated by upper endoscopy (*n* = 1), and CT (*n* = 1).

GNECs (*n* = 3): Subjects were evaluated by upper endoscopy (*n* = 2), CT (*n* = 2), ^68^Ga-DOTA-TATE PET/CT (*n* = 3), ^18^F-FDG PET/CT (*n* = 1).

### Histological diagnosis

All biopsy/surgical specimens were evaluated (H&E, immunohistochemistry) and reviewed by an independent expert NEN pathologist (WZ) and reported in accordance to WHO 2017 and TNM 8th edition classifications for the foregut (gastric) neuroendocrine neoplasms [1, 26–29].

### NETest blood sample collection

Peripheral blood samples (3 ml) were collected in EDTA tubes, mixed, and stored on ice. Tubes were anonymously coded and stored at − 80 °C within 2 h of collection per standard molecular diagnostics protocols for PCR-based studies [30]. Randomly selected, coded blood samples were sent de-identified to Wren Laboratories LLC, Connecticut, USA for NETest measurement.

### NETest measurement

Details of the PCR methodology, mathematical analysis and validation have been published in detail, comprising a 2-step protocol (RNA isolation/cDNA production and qPCR) from EDTA-collected whole blood [11, 18, 30]. The assay was undertaken in a USA clinically certified laboratory (Wren Laboratories CL-0704, CLIA 07D2081388). Transcripts (mRNA) were isolated from EDTA-collected whole blood samples (mini blood kit, Qiagen, Valencia CA) and real-time PCR performed on pre-spotted plates. Target transcript levels were normalized and quantified versus a population control [11, 18, 30]. Final results are expressed as an activity index (NETest score) from 0 to 100% [11, 18, 30]. NETest-positive: ≥20.

### Statistical analysis

The required total sample size (NETs and controls, power 0.8 and α = 0.05, two independent study groups, continuous primary endpoint = based on published NETest results in NETs (mean/SD) vs. controls (mean) [11, 18, 30] was calculated to be a minimum of 40 patients/subjects in each group. Intergroup analyses were undertaken using 2-tailed non-parametric tests (Mann-Whitney U test). Area under the Receiver Operator Characteristic (AUROC) analysis was used to determine the diagnostic accuracy of the NETest [24, 31, 32]. Metrics calculated included sensitivity and specificity. Prism 7.0 for Windows (GraphPad Software, La Jolla California USA, www.graphpad.com) and MedCalc Statistical Software version 16.2.1 (MedCalc Software bvba, Ostend, Belgium; http://www.medcalc.org; 2017) were utilized. Statistical significance was defined at a *p* value < 0.05. Data are presented as mean ± SD. Age at blood draw or diagnosis, follow-up time (FU), and biochemical parameters were presented as mean and range.

## Results

The study results are reported according to the Standards for Reporting of Diagnostic Accuracy (STARD) [25] (Fig. 1, STARD Flow chart).

### Disease extent

#### Image-positive (macroscopic disease)

Macroscopic disease (lesions detectable on gastroscopy) was evident in sixteen: lesions size: ≤5 mm (50%), > 5–9.9 mm (17%), 10–19.9 mm (17%), ≥20 mm (17%) [≥10 mm: 34%]. Lesions were multiple: 38%, single: 62%, submucosal: 13%, or ulcerated: 13%. NET diagnosis was confirmed in all based on biopsy and histological evaluation. All were well-differentiated: NET G1, *n* = 14; NET G2, *n* = 1; NET G3, *n* = 1; and comprised Type 1 GNETs, *n* = 15 or Type 3, *n* = 1. CT examinations were available in nine, and in one - a CT and MRI. Anatomical imaging (CT/MRI) was undertaken within 4.6 months (range: 1-12) from the NETest blood draw. In five cases (4 with CT available and 1 with CT and MRI), abnormal findings e.g., thickening of the gastric wall, were identified. Follow-up gastroscopy findings confirmed the imaging data – these ranged from ulceration and submucosal lesions to multiple polyps throughout the entire stomach. A ^68^Ga-DOTA-TATE PET/CT was available in 10 (performed within 5.7 months [range: 1-12] of the blood draw), and in 5, an increased tracer uptake in the stomach was reported. Typically, this ranged from multiple, focal areas of increased tracer uptake correlating with the wall thickening to uptake in nodular lesions with SUV_max_ (as high as 28.8, in one instance). EUS was performed in 9 for locoregional disease evaluation and assessment of lesions invasion in pre-treatment planning and confirmed the above-described findings.

#### Image-negative

Thirty GNENs were image-negative (with no detectable signs of recurrence or metastatic disease) on a follow-up gastroscopy (*n* = 25), EUS (*n* = 6), CT (*n* = 21), ^68^Ga-DOTATATE PET/CT (*n* = 17), or ^18^F-FDG PET/CT (*n* = 2; GNET Type 1 G2, Ki-67 15%, and GNEC, Ki-67 75%). Twenty-seven were well-differentiated GNETs Type 1: NET G1, *n* = 18; NET G2, *n* = 9; and three were poorly-differentiated NECs.

#### Image- and histology-negative

Amongst thirty image-negative, 21 subjects (NET G1, *n* = 10; NET G2, *n* = 8; NEC, *n* = 3) were margin-negative post-last polypectomy/gastric biopsy (*n* = 16) (amongst these, eight had undergone a partial gastrectomy), and five underwent total gastrectomy. CT was available and always negative in 15, ^68^Ga-DOTA-TATE PET/CT in 13 (all negative), ^18^F-FDG PET/CT in 1 (GNEC, Ki-67 75%) (negative), and EUS in 6 (all negative).

#### Image-negative and histology-positive

There were nine image-negative GNETs (all Type 1) who tested positive on histological examination despite no detectable signs of recurrence or metastatic disease on a follow-up gastroscopy (*n* = 9), CT (*n* = 6), ^68^Ga-DOTA-TATE PET/CT (*n* = 5), and ^18^F-FDG PET/CT (*n* = 1). Five had a post-polypectomy positive resection margin, and four were positive on random biopsies of the gastric mucosa taken at follow-up gastroscopy.

### Diagnosis

**Fig. 2:**
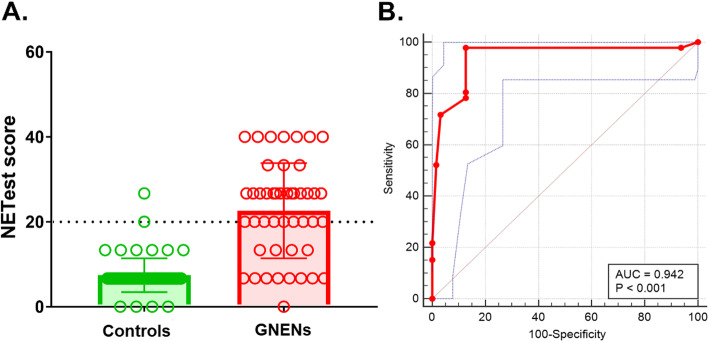
NETest levels in GNENs and controls. **a** NETest measurements were significantly higher in GNENs (23 ± 11; *n* = 46) compared to controls (7 ± 4, *p* < 0.0001; *n* = 63). Mean ± SD. *Dotted line*: NETest upper limit of normal (20%). **b** The AUROC for NETest levels in GNENs and controls: The AUROC (*red line*) for differentiating NENs from controls was 0.94 (95%CI: 0.88 to 0.98, *p* < 0.0001). A maximum AUC = 1 identifies an ideal (perfect) differentiation between disease and non-disease subjects. The diagonal line (AUC = 0.5) corresponds to chance discrimination. The NETest AUC > 0.9 (*red line*) indicates that it is an excellent biomarker for GNEN

**Fig. 3:**
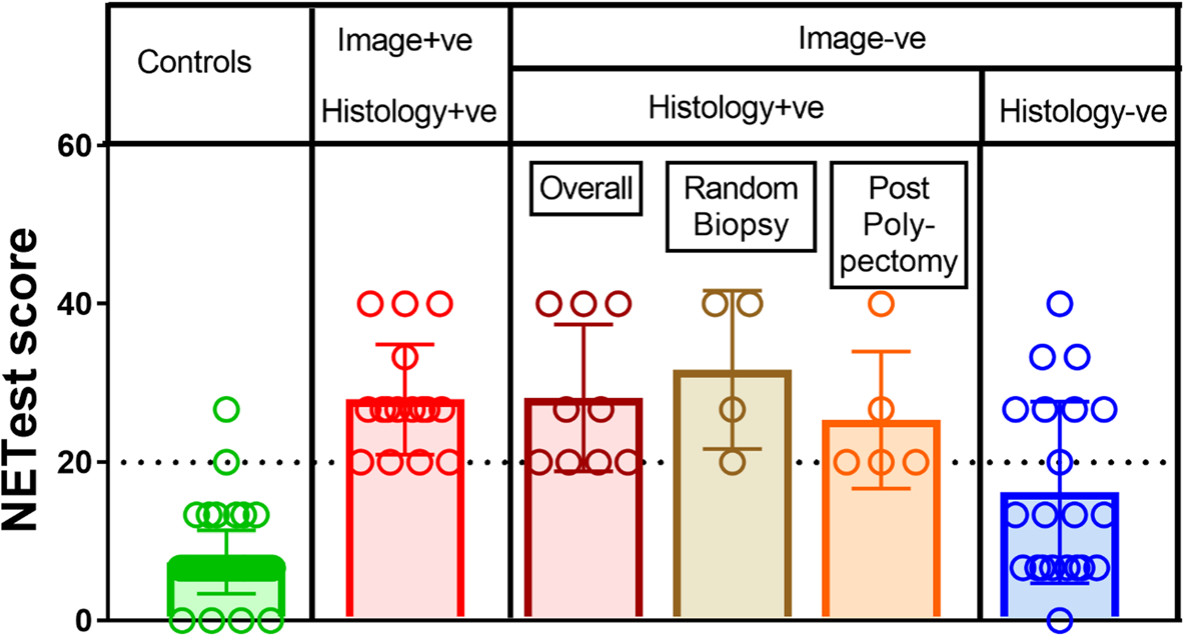
NETest levels in GNENs comparing image-positive and –negative disease. The NETest was significantly higher in image-positive (gastroscopy and anatomical and/or functional imaging) disease (28 ± 7) compared to controls (7 ± 4, *p* < 0.0001). This was significantly increased compared to levels in image-negative (gastroscopy and anatomical and/or functional imaging) and histology-negative subjects (16 ± 11, *p* = 0.002). NETest levels in image-positive and histology-positive disease (28 ± 7) were not different to histology-positive but image-negative disease (28 ± 9, *p* = ns). In the histology-positive disease, the NETest scores reflected low disease activity (stable disease). Mean ± SD. Dotted line: NETest upper limit of normal (20%)

**Fig. 4:**
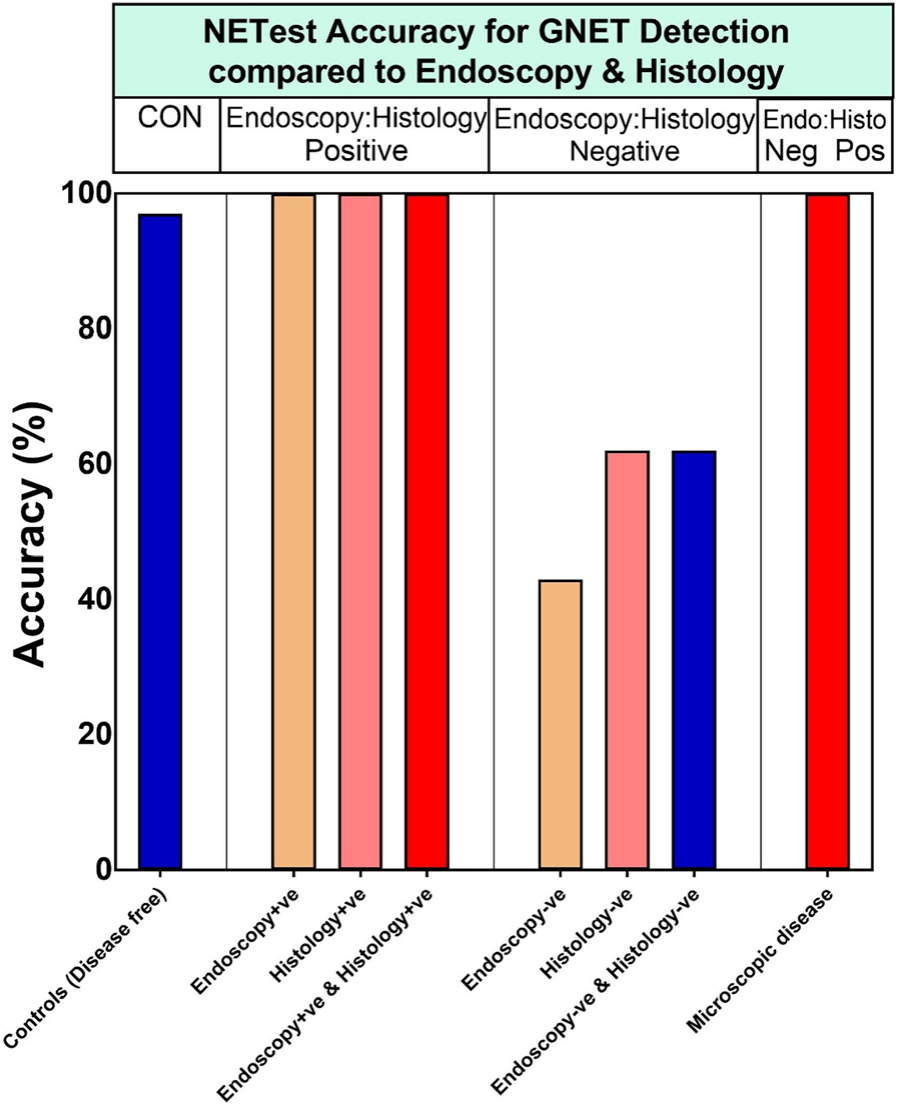
The NETest accuracy in Gastric NEN identification. The NETest was 97% accurate (normal score 61/ 63) in controls (the two elevated NETest scores were 20% [borderline] and 27%, in two women aged 28 and 42 with no history of co-morbidities, or medications). NETest score was 100% (16/16) accurate for the identification of histologically confirmed macroscopic disease (both endoscopy and histology-positive). Twenty-five cases were histology-positive (16 with macroscopic and 9 microscopic disease). In all (25/25), the NETest was positive. Thirty subjects were endoscopy-negative (no detectable lesions). In these, the NETest was within normal range in 43% (13/30); but nine had a microscopic disease histologically confirmed. Twenty-one individuals were negative on last histological examination (negative polypectomy margin, or gastric biopsy, or post total-gastrectomy); 13 had NETest within normal range (62%). Twenty-one subjects had no detectable lesions by gastroscopy and tested negative for NEN on last histological examination, thirteen were NETest-negative (62%). The NETest was 100% accurate for identifying microscopic disease (9/9) (no lesions detectable on gastroscopy, but confirmed by histology [positive]: 5 with positive polypectomy margin and 4 positive on random gastric biopsy). Abbreviations: endoscopy-ve = endoscopy-negative; endoscopy+ve = endoscopy-positive; histology-ve = histology-negative; histology+ve = histology-positive

NETest was increased in GNENs (23 ± 11) vs controls (7 ± 4, *p* < 0.0001) (Fig. 2). For macroscopic disease (*n* = 16), the NETest was 100% accurate (16/16) in identifying disease. In image-negative but histology-positive (*n* = 9) patients, the NETest was positive in all (9/9). The NETest levels in macroscopic disease (28 ± 9) were significantly higher than in controls (7 ± 4, *p* < 0.0001), or in those who were considered disease-free (19 ± 11, *p* = 0.02) (Fig. 3). The latter included: negative polypectomy margin, no imaging detectable lesions and/or testing negative for NET on a gastric mucosa biopsy. Levels in macroscopic disease, however, were not different to the nine patients with microscopic disease (histologically confirmed as a positive polypectomy margin or on random gastric biopsy without gastroscopy detectable lesions/polyps) (24 ± 10, *p* = 0.5). In the image-negative/R0 cohort (IND-R0, *n* = 21), the NETest was elevated in eight (1 NEC and 7 GNETs Type 1: 5 NET G2, 2 NET G1; 2 NET G2 had a history of lymph node metastases, and all 7 GNETs Type 1 exhibited chronic atrophic gastritis and hypergastrinemia off PPI). Overall, the diagnostic metrics for the NETest were: accuracy (90%), sensitivity (100%) and specificity (87%) (Fig. 4). The AUROC to differentiate GNENs from controls was 0.94 (95%CI: 0.88 to 0.98, *p* < 0.0001) (Fig. 2b).

No correlation was found between lesion size as identified on gastroscopy, or Ki-67 (*p* > 0.05), and NETest levels. The number of subjects (*n* = 16) in the macroscopically detectable disease subgroup, however, may not be sufficient to rigorously define the relationship between these characteristics.

### Impact of surgery on the NETest scores

#### Total gastrectomy

Prior to referral to our center, five subjects underwent total gastrectomy (2 GNECs and 3 GNETs Type 1). All were image-negative (gastroscopy, *n* = 3; CT, *n* = 3; ^68^Ga-DOTA-TATE PET/CT, *n* = 4) at blood draw. Mean follow-up since surgery was 1.9 years (0.6–3.2). In two subjects (one GNEC [FU 1.65 years] and one GNET Type 1 [FU 1.9 years]), the NETest was within normal range, while three (one GNEC [Ki-67 50%, FU 2.3 years] and 2 GNETs Type 1 [FU 3.2 years and 7 months]) were NETest-positive (Table 2).

**Table 2:** Characteristics of post-total gastrectomy NETest-positive subjects (*n* = 3)

Subject #	GNEN Type	Surgical Pathology	Grade (Ki-67)	Post-surgery blood (years)	NETest score	Endoscopy^a^	CT^a^	^**68**^Ga-SSA PET/CT^a^
1	GNEC	pT1N0	G3 (50%)	2.3	27%	1 month: Negative^b^	2 years: Negative^b^	2 months: Negative^b^
2	1	pT2mN1	G2 (10%)	3.2	27%	1.8 years: Negative^b^	0 month: Negative^b^	9 months: Negative^b^
3	1	pT2mN1	G2 (3%)	0.65	40%	ND	ND	0 month: Negative^b^

ND = not done/no data; ^a^ time between imaging (endoscopy, anatomical, functional) and blood draw for the NETest; Negative^b^ = Negative for NET; 0 month = blood taken just prior to imaging; Subject # = Subject number

#### Partial gastrectomy

Eight subjects underwent partial gastrectomy (mean FU since surgery 4.6 years [0.1–10.5]), all were margin-negative after last polypectomy/on biopsy. Five were NETest-negative and three NETest-positive (Table 3). The latter were all GNETs Type 1 G2, with active CAG and elevated gastrin levels without PPI administration.

**Table 3:** Characteristics of post-partial gastrectomy NETest-positive subjects (*n* = 3)

Subject #	GNET Type	Gastrectomy Type	Surgical Pathology	Grade (Ki-67)	Post-surgery blood (years)	NETest score	Endoscopy prior to blood draw^a^	Previous Endoscopy^b^	FU-imaging^c^
4	1	Wedge gastric body resection	pT1	G2 (3%)	1.9	27%	2 months: Negative^d^	10 months: Foci of NE cells	8 months: Increased tracer uptake on ^68^Ga-SSA PET/CT in pancreatic head and duodenum CT-negative^d^ EUS: ND
5	1	Wedge gastric body resection	pT1	G2 (3%)	5.3	20%	15 months: Negative^d^	3 years: Negative^d^	3 months: CT-negative^d^
6	1	Pylorus preserving gastrectomy	ND	G2 (10%)	3.9	33%	17 months: Negative^d^	4 months: 5 recurrent NET lesions	0 month: CT-negative^d^

^a^Gastroscopy prior to blood draw (time between endoscopy and blood draw for the NETest); ^b^Previous Endoscopy (time between two last endoscopies); ^c^Follow-up (FU) imaging (time between imaging and blood draw); 0 month = blood taken just prior to imaging; Negative^d^ = Negative for NET; ND = not done/no data; NE = neuroendocrine; Subject # = Subject number

### Residual disease identification

Nine GNETs were image-negative with no macroscopically detectable lesions on a follow-up gastroscopy (*n* = 9), or CT (*n* = 6), ^68^Ga-DOTATATE PET/CT (*n* = 5), and ^18^F-FDG PET/CT (*n* = 1), but were positive on histological examination.

#### Positive polypectomy margin

Five subjects had a positive polypectomy margin. In all (5/5) the NETest was elevated (25 ± 9).

#### Positive blind biopsy

Four GNETs (Table 4), without macroscopically detectable lesions, had random biopsies of the gastric mucosa taken. All (4/4) exhibited histological features of microscopic tumor. The NETest was elevated in all (32 ± 10).

**Table 4:** Characteristics of random-biopsy and NETest-positive subjects (*n* = 4)

Subject #	GNET Type	Histology	Ki-67	NETest score	Endoscopy-blood^a^ (months)
7	1	Chronic atrophic gastritis with intestinal metaplasia. Dispersed nests of cells with neuroendocrine differentiation, diffuse submucosal infiltration.	< 1%	27%	4
8	1	Nests of neuroendocrine cells: 3.5 mm infiltration.	2%	40%	12
9	1	NET G2, and 2 mm infiltration evident.	3% (14.5% prior)	40%	22
10	1	Multiple NET foci.	1%	20%	ND

ND = not done/no data; Endoscopy-blood^a^ = Time between endoscopy and blood draw for the NETest; Subject # = Subject number

## Discussion

GNENs represent a heterogeneous group of neoplasms which either arise asymptomatically or present with non-specific upper gastrointestinal symptoms. With the increasingly widespread use of endoscopy, GNENs are now diagnosed not only with rising frequency but also at an earlier age. In spite of their indolent behavior, an early diagnosis is important to facilitate the feasibility of curative resection [33]. It is also important for defining the biology of these lesions, and initiating monitoring as micronodules may grow or recur over time. In a prospective study, approximately 64% of Type 1 GNETs recurred in a median of 8 months after endoscopic resection [34]. Sixty-seven percent of these thereafter experienced a second recurrence within 8 months [34]. Metastatic potential has been correlated with tumor size [1, 4, 7]. However small (minute) lesions with metastatic spread have been reported [35]. The clinical impact of this is not known yet [36]. Other features of a more aggressive potential have been attributed to grade, invasion beyond the mucosa layer, lymph node involvement or early recurrence after endoscopic polypectomy [33, 37]. Given the inability to accurately define the propensity of a GNET to progress, a careful assessment of all GNETs is required to determine the most appropriate treatment. This involves either resection or ongoing surveillance [1, 8]. Current management monitoring strategies are focused on annual surveillance endoscopy for life [1, 27, 38, 39]. Gastroscopy and multiple biopsies provide the principal tools for diagnostic workup and surveillance programs.

According to NCCN guidelines [39], Type 1 GNETs < 20 mm, should be followed up with endoscopy every 6–12 months after treatment for the first 3 years; and beyond three years - annually. Patients with Type 3 tumors or large (> 20 mm) Type 1 lesions, are recommended to be followed-up every 3 to 12 months following resection, and every 6 to 12 months thereafter. Imaging such as CT or MRI are used depending on clinical indications [39]. The ENETS guidelines [1, 38] recommend endoscopic follow-up every 12 months for recurrent Type 1 tumors, and every 24 months for individuals without recurrence. The post-gastrectomy follow-up for GNECs is similar to that for gastric adenocarcinoma [40].

Repeat gastroscopies with biopsies, however, are invasive, and associated with an increased risk of bleeding or perforation, are costly, and engender substantial patient discomfort and work absence. As a consequence, compliance becomes an issue. In our cohort, 29% had a follow-up gastroscopy for a period greater than 24 months (range 25 months to 11 years). Given the adverse medical and economic logistics of repetitive interventions, the availability of a circulating biomarker that is related to the tumor biology, has considerable advantages.

The ability to identify asymptomatic/non-specifically presenting disease (in an at risk population), or increased biological activity of the disease (its aggressiveness and progress) using a blood-based assay, has clinical significant relevance. Of particular applicability would be use of such a test in individuals at an increased risk of developing GNETs, such as e.g. pernicious anemia (risks range from 2 to 9% [2] to 58% [41, 42]). Seventeen percent of our cohort were diagnosed with anemia and vitamin B12 deficiency: 30% of these exhibited pernicious anemia, 43% other autoimmune diseases (Hashimoto thyroiditis, myasthenia, Graves’ disease), and in the remainder, the cause for vitamin B12 deficiency was not specified.

The development of a multianalyte transcriptomic signature (the NETest) for neuroendocrine tumors and the encouraging reports of its efficacy have been documented in enteropancreatic NETs [11–17] and lung NETs [18–20]. The accuracy for the NETest diagnosing these tumors is 93–97% [12, 16, 17], metrics which meet the NIH criteria of an optimal diagnostic biomarker [43]. Although NETs represent a heterogeneous group of tumors, they share a common genotype and it is plausible that the NETest signature, because it is based on gene expression measurements, would be effective and accurate for identifying tumors from other organ sites in which tumors develop. To date, ten GNET patients in diverse studies, have been investigated by the NETest assay [11, 12, 15–17, 23, 24]. These included both G2 and G3 GNETs; all were NETest-positive and CgA-negative [24]. In a surgical cohort, a Type 3 GNET (Ki-67 25%) pre-operatively exhibited a high NETest score (93%). After the patient underwent complete tumor resection, she developed loco-regional recurrence at 6 months post-surgery. Of note, the NETest was elevated as early as one month after surgery. The test predicted recurrence which was related to the biology of the lesion [15]. Other reported GNETs were a part of larger NET cohorts in which the NETest was evaluated as a predictor of somatostatin analogue efficacy [11], or when an independent validation was undertaken [17]. All exhibited an increased NETest score suggesting that the signature identified GNETs.

Based upon these observations, we undertook the current study to evaluate the NETest assay in an independent, large GNEN cohort comprising Type 1 (majority), NECs and a Type 3 NET G3. The NETest diagnostic metrics were: 90% accuracy, 100% sensitiity, and 87% specificity. The “lower” specificity is probably accounted for by the small number of image- and histology-negative subjects which were NETest-positive. It is likely that the increased NETest scores represent the process of ECL transformation into a GNET based upon the underlying CAG and the subsequent hypergatrinemic drive. Nevertheless, the AUC for differentiating a GNEN from controls was 0.94 (*p* < 0.0001); an AUC ≥0.9 is considered scientifically to represent an excellent biomarker [44].

Since the diagnostic metrics met the NIH criteria for diagnostic usage, we then evaluated the test accuracy for identifing macroscopic versus microscopic disease. The key issue was to assess its utility in identifying residual/recurrent disease. Identification of residual disease is a clinically important issue as its presence mandates the need for further treatment (endoscopic mucosal or submucosal resection, or ablative surgical procedures) [1]. In macroscopic disease, the NETest was positive in all cases. All polyps ≥10 mm (34% of the image-positive cohort) (minimum size recommended for resection [1]), were 100% NETest positive. Moreover, the NETest was 100% sensitive for identifying small lesions < 5 mm. These comprised 50% of the macroscopically detectable disease. Overall, this 100% accuracy represents important adjunctive clinical information for the recommendation of endoscopic resection. While NETest levels did not correlate with polyp size per se, it is likely that using molecular biological criteria as opposed to size may in the future provide a balanced, scientific basis for guiding the need for resection.

In the image-negative (macroscopically undetectable lesions) but histology-positive (microscopic residual/recurrent disease), the NETest was positive in 100% (9/9). These comprised five subjects with positive polypectomy margin and four cases in which random biopsies of the gastric mucosa were undertaken (as recommended [1] e.g. due to CAG and increased risk of dysplasia). The levels of the NETest were not different between these two subgroups (25 ± 9 vs 32 ± 10, *p* = NS). For the 4 random-biopsy positive subjects, even neuroendocrine infiltrations as small as 2 mm (Subject #9) were associated with a positive NETest score. These data are consistent with the known sensitivity of molecular genomic analysis in the identification of microscopic recurrent or residual NET disease [45].

Identifying patients with residual disease is a critical clinical issue since under such circumstances, wide local excision or partial gastrectomy should be considered [1]. After excision, the endoscopic follow-up is recommended, but the most appropriate timing has never been defined, although most consider annual or biannual re-endoscopy as prudent [1, 38, 39]. When mucosectomy techniques and follow-up programs are undertaken, a recurrence free survival of 24 months can be attained, with the overall excellent prognosis of Type 1 GNETs [1].

In our cohort, thirteen subjects underwent GNEN surgery (median 2015 [range 2006–2017]) for, either Type 1 or 3, prior to tertiary medical center referral. In these, we investigated the impact of the surgery/resection on the NETest scores. Five subjects underwent total gastrectomy and eight – partial gastrectomy. Three of the 5 total gastrectomy subjects were NETest-positive (scores: 27–40). All were ^68^Ga-DOTATATE PET/CT-negative. Two of these subjects (#2 & 3) had positive lymph node metastasis at the time of the initial operation. It seems probable that this represents disseminated disease. In two patients, surgery occurred more than 2 years prior to the blood test. It seems likely that the low positive scores in each (NETest 27) reflects microscopic disease recurrence not yet detectable by imaging. These data are consistent with previous reports that NETest positivity can precede image-based disease identification by 1–2 years [11, 30]. It is likely that with time NETest levels will steadily increase up to the point where disease burden is sufficiently large for standard imaging or functional imaging to identify [40].

None of the surgical patients underwent an antrectomy alone. Eight had a partial gastrectomy either: trans-hiatus lower esophagus and upper gastric resection, wedge gastric fundus or body resection, Billroth Type I or pylorus preserving gastrectomy. Each of these patients were margin-negative at their final polypectomy/biopsy follow-up. Out of these eight, three were NETest-positive. All three were GNET Type 1 G2, with active CAG and elevated CgA and gastrin levels without PPI administration. Two underwent wedge gastric body resection, and one pylorus preserving gastrectomy, thus most likely had antral G-cell secreting remnant. We believe that an elevated NETest after resection of the Type 1 GNETs (endoscopic/surgical) probably represents the ongoing pathobiological process whereby the gastrin drive for ECL transformation to GNETs persists despite either surgical antrectomy [46], or other partial gastrectomies to reduce ECL cells [47]. Even with an antrectomy, duodenal gastrin secretion is maintained and represents up to 20% of circulating gastrin levels [46]. It is noteworthy that in 50% undergoing laparoscopic antrectomy. A previous report identified no regression of ECL cell numbers (hyperplasia/foci of cells) was noted in 50% undergoing laparoscopic antrectomy [48]. Under such circumstances, a continued gastrin drive could result in microscopic foci of ECL neoplasia that subsequently manifest as an overt GNET [8]. Subject #6 classically exemplifies this with 5 lesions developing ~ 2 years after pylorus preserving gastrectomy. It is for these reasons, that a subtotal or total gastrectomy may represent a more suitable options than antrectomy. Subtotal gastrectomy facilitates more extensive removal of G cells, while total gastrectomy is reserved for those cases with diffuse and substantial disease in the gastric fundus [49].

**Fig. 5:**
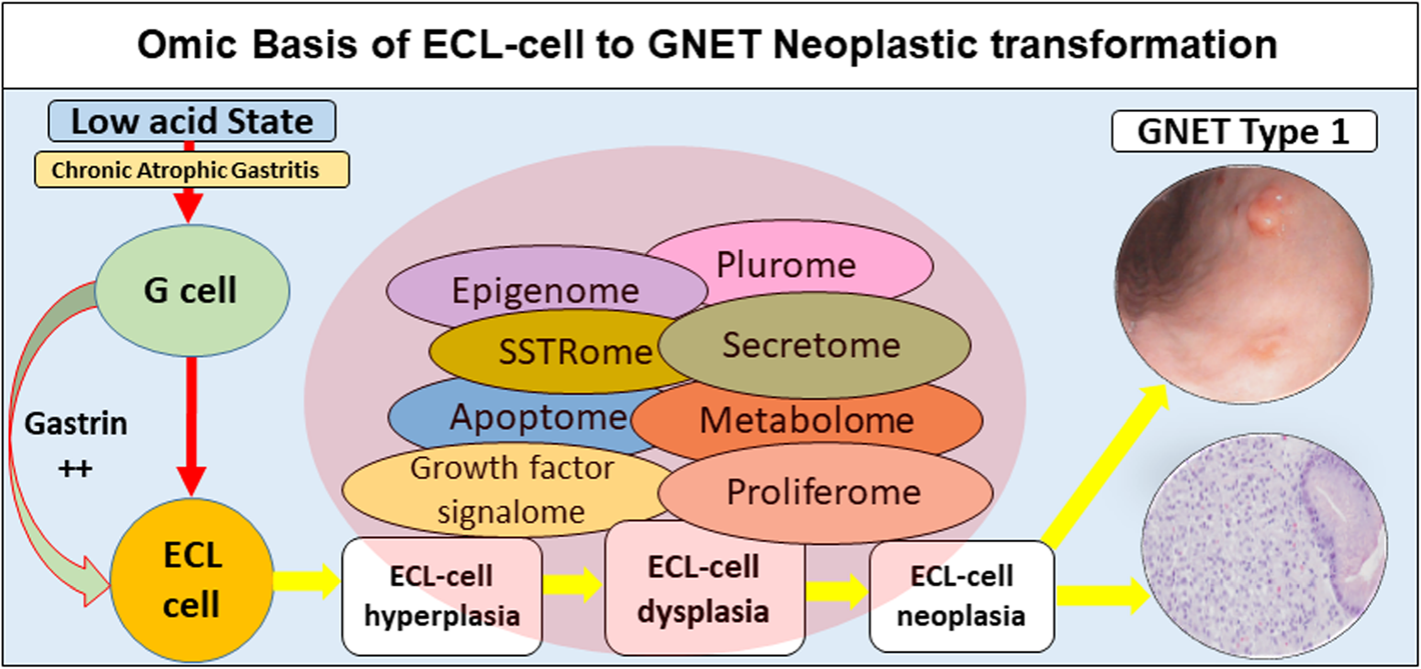
Omic regulated Hypergastrinemic transformation of the ECL cells to GNETs Type 1. The loss of parietal cell function (chronic atrophic gastritis [CAG], pernicious anemia, or other autoimmune diseases) is associated with a low acid state, gastric pH increase and consequent hypergastrinemia with G cell hyperplasia. Since gastrin is a trophic agent for ECL cell proliferation, sustained hypergastrinemia results in ECL cell transformation from hyperplasia to dysplasia, and thereafter neoplastic transformation to a Type 1 GNET (*insets right: endoscopy (superior) and H&E microscopy (inferior)*). The canonical molecular drivers of neuroendocrine tumorigenesis (*red central circle*) include a number of *omes*, e.g. proliferome, growth factor signalome, metabolome, apoptome, etc. The NETest is a multigene assay designed to measure the individual “omes” and identify their expression in blood. Endoscopic and histological images adapted with permission from Lanke et al. [51]

The NETest signature in blood therefore provides an extremely sensitive index to monitor and identify in a real-time the biology and activity of otherwise undetectable disease [45]. Differential expression of 51 NET marker genes in peripheral blood which includes measurements of biologically relevant genes that constitute the different “omes” (SSTRome, proliferome, metabolome, secretome, epigenome, and plurome), are more likely to differentiate progressive disease (PD) from stable disease (SD) [50] (≥80 vs ≤40) [16]. In a recently published US registry study which enrolled 68% GEP-NETs, SD was associated with a low NETest (≤40%) in 87% of patients (54/62); while PD was associated with a high score (≥80%) in 81% of patients (21/26) [12]. In the current study cohort, the NETest was low, ≤40% in all cases, in line with an indolent, slow-growing disease, which at the time of blood draw, was localized (amongst Type 1 GNETs, three individuals had a history of lymph node metastasis, but at the time of blood draw, none had metastases detectable by imaging), or were image-negative. In the GNENs G3 subgroup, no individuals exhibited PD, clinically or by imaging. All NECs were image-negative and had NETest scores < 40. The subject with disease in situ (3 cm polypoid lesion, stable over 7 months by both gastroscopy and CT) exhibited a NETest score of 20. We note one previously published GNET Type 3 grade 3 [15] which exhibited a score of 93. This was clearly a highly aggressive disease as the patient developed loco-regional recurrence within 6 months of surgery. These data support the concept that an omic evaluation will likely be useful in defining the biology and future clinical trajectory of these tumors. In the future, using prospective study tools, we envisage a more sophisticated version of the NETest. This would provide additional information based on quantification of specific biologically relevant gene clusters or “omes” e.g., proliferome, that provide more precise genomic information regarding the propensity of such lesions to progress. Moreover, it is likely that such information could be used to define the likelihood of a gastrin-induced ECL cell hyperplasia transforming into a neoplasia (Fig. 5).

There are a number of limitations in the study. A positive NETest was evident in 3% (2 out of 63) of controls. We are unable to evaluate whether these are false or true positive as none underwent upper endoscopy and no biomarker studies e.g., gastrin were undertaken in them. Furthermore, the majority of the NEN study cohort comprised Type 1 GNETs. This, however, is consistent with the epidemiology of these tumors; the imbalance in numbers for different subtypes is therefore consistent with real-world experience. Interpretation of some of the results, in particular the image-negative but NETest-positive cases, however, was hindered by the lack of contemporaneous imaging. Additionally, all the endoscopic assessments were not undertaken physically in our Centre due to geographic and health economy reasons, thus this could contribute to some variability in result reporting. It should be noted that the study was based upon real-world principles and many GNETs, because of their presumed indolence and low malignant potential, are not closely monitored and less commonly undergo functional scanning as in the stringent protocols for small intestine or pancreatic NETs. Optimally, a prospective study would allow clearer understanding of at what point in time blood gene level elevations would warrant intervention. Moreover, a broader evaluation of the NETest in Type 3 NETs and GNECs would provide further information to define this aggressive subgroup of neoplasia. These NEC-equivalent lesions might benefit from the availability of more sensitive monitoring strategy rather than endoscopy and biopsy alone. Nevertheless, it is clear that the test accurately identifies GNENs (both Type 1 and 3) and provides a basis for the use of non-invasive monitoring to provide adjunctive information as to the clinical status of GNENs.

## Conclusions

The NETest has been independently validated in a substantial GNET cohort and identified to function as an in vitro diagnostic for GNETs. Elevated levels identified both macroscopic and microscopic residual disease. The availability of a blood test that is as effective as histology and more sensitive than imaging modalities (endoscopy/anatomical or functional) provides a clinically useful adjunct to the life-long monitoring strategy. The safety, comfort and cost implications for diminishing the extent of endoscopic surveillance also have obvious patient and health economic advantages, but this requires a formal study. A blood-based multigene real-time assessment of histology/image-negative disease provides the opportunity to identify and monitor disease from a previously imperceptible time point. We propose that the future strategy of molecular genomic assessment of GNETs should focus on the identification of *omic* clusters levels that specifically define hypergastrinemia-induced neoplastic transformation of ECL cells. Such a refined tool would provide a predictive goal to identify when dysplasia has transformed into neoplasia and define when a GNET might actually require resection.

## Data Availability

The datasets used and/or analyzed during the current study are available from the corresponding author on reasonable request.

## Abbreviations

AUROC: Area under the Receiver Operating Characteristic
CAG: Chronic atrophic gastritis
CgA: Chromogranin A
ECL: Enterochromaffin-like
GNEN: Gastric neuroendocrine neoplasm
GNET: Gastric neuroendocrine tumor
IND: Image-negative disease
IPD: Image-positive disease
NEC: Neuroendocrine carcinoma
NEN: Neuroendocrine neoplasm
NET: Neuroendocrine tumor
NS: Not significant
PD: Progressive disease
R: Resection margin
SD: Stable disease
SSA: Somatostatin analog
